# Bisphenol S and Its Chlorinated Derivatives in Indoor Dust and Human Exposure

**DOI:** 10.3390/toxics12070448

**Published:** 2024-06-21

**Authors:** Yi Qian, Jianqiang Zhu, Ruyue Guo, Hangbiao Jin

**Affiliations:** 1 Department of Environmental Engineering, Taizhou University, Taizhou 318000, China; 2 Key Laboratory of Microbial Technology for Industrial Pollution Control of Zhejiang Province, College of Environment, Zhejiang University of Technology, Hangzhou 310032, China

**Keywords:** BPS, chlorinated BPS, inhalation exposure, indoor dust, human intake

## Abstract

Bisphenol S (BPS), an environmental endocrine disruptor, has been identified in global environmental matrices. Nevertheless, limited studies have investigated the presence of chlorinated analogues of BPS (Clx-BPSs) with potential estrogenic activities in environmental matrices. In this study, the occurrence of BPS and five types of Clx-BPSs was characterized in indoor dust (*n* = 178) from Hangzhou City. BPS was measurable in 94% of indoor dust samples, with an average level of 0.63 μg/g (<LD–2.4 μg/g). Among the detected Clx-BPSs homologues, Cl_1_-BPS (2-chloro-4-(4-hydroxyphenyl)sulfonylphenol; detection frequency 70%), Cl_2_-BPS-2 (2-chloro-4-(3-chloro-4-hydroxyphenyl)sulfonylphenol; 65%), and Cl_2_-BPS-1 (2,6-dichloro-4-(4-hydroxyphenyl)sulfonylphenol; 61%) were among the frequently detected Clx-BPSs. Cl_1_-BPS was the most abundant analyte, with an average of 0.048 μg/g (<LD—0.24 μg/g), followed by Cl_2_-BPS-1 (0.035 μg/g, <LD—0.14 μg/g), and Cl_2_-BPS-2 (0.031 μg/g, <LD—0.13 μg/g). Significant correlations in indoor dust concentrations were observed between BPS and Cl_1_-BPS (*p* < 0.01), as well as between BPS and Cl_2_-BPS-1 (*p* < 0.01). Moreover, an estimation was made for the total daily intake of Clx-BPSs via the ingestion of indoor dust by infants, children, and adults. This study presents the first evidence of the existence of Clx-BPSs in indoor dust, concurrently highlighting the necessity to address their potential human exposure risks.

## 1. Introduction

Bisphenol S (4, 4′-sulfonyldiphenol; BPS), consisting of two hydroxyphenyl groups connected by a sulfone linkage, belongs to an artificial chemical with a large worldwide production volume [1,2,3]. It has exceptional resistance to light, high temperature, and oxidation [4]. Owing to these properties, BPS has been widely applied as an additive agent in the manufacturing of polycarbonate plastics, adhesives, dyes, epoxy resins, and plastic coatings [5]. Notably, BPS has also emerged as a prominent alternative to bisphenol A, which faced global restrictions and bans because of its safety concerns [6,7]. Many consumer products (such as plastic food and beverage containers, baby feeding bottles, furniture, and paper products) contain BPS [2,8]. In addition, BPS has become ubiquitous in the global environment, including surface water, drinking water, indoor dust, air, and wild animals [2,3,9,10,11]. Therefore, the general population worldwide is widely exposed to BPS through various pathways [12]. Studies have reported that BPS could potentially pose equal or greater harm than BPA in certain aspects [13,14]. For instance, BPS demonstrates qualitatively similar impacts on the estrogen and androgen receptor functions compared with BPA [15]. Furthermore, additional adverse health effects have been observed to be due to BPS exposure, including associations with obesity and developmental defects [16,17].

Chlorinated derivatives of BPS, denoted as Clx-BPSs, are formed by replacing hydrogen atoms on the phenyl ring of BPS with varying numbers of chlorine atoms [18]. Clx-BPSs mainly include monochloro-BPS, dichloro-BPS, and trichloro-BPS [18]. Several kinds of Clx-BPSs homologues, such as 2-chloro-4-(4-hydroxyphenyl)sulfonylphenol (Cl_1_-BPS) and 2,6-dichloro-4-(4-hydroxyphenyl)sulfonylphenol (Cl_2_-BPS-1), have been reported to exist in various paper products [18,19,20]. Therefore, human exposure to Clx-BPSs is expected to occur through dermal contact when handling. In addition, humans may ingest Clx-BPSs through dietary intake and inhalation, as indicated by data reported on BPS [4,21]. However, in-vitro evidence revealed that the estrogenic activity of Clx-BPSs elevated with the degree of chlorination [20]. Furthermore, Clx-BPSs exhibited the peroxisome proliferator-activated receptor-γ (PPARγ) activity, which is enhanced as the number of substituted chlorine atoms increased [22]. Hence, it is imperative to assess human exposure to these emerging pollutants thoroughly.

Indoor dust pollution is significantly associated with human health [23]. Indoor dust may contain various kinds of toxic substances (including toxic metals, herbicides, pesticides, plastics, and other organic pollutants) [24,25,26,27], posing long-term human health risks upon ingestion [28,29]. Children and infants are particularly susceptible to the adverse effects of indoor dust inhalation because of their high sensitivity to pollutants [30,31]. Pollutants observed in indoor dust originate from outdoor dust infiltration, atmospheric deposition, human activities, and household furnishings [32,33]. Many previous studies have characterized the existence of BPS in global indoor dust matrices [34]. A recent study has confirmed the existence of Clx-BPSs in paper products, including thermal paper, household paper, and corrugated boxes [18]. However, to our knowledge, studies investigating the occurrence of Clx-BPSs in various dust matrices remain lacking.

In the present study, samples of indoor dust were collected from 178 distinct residential apartments within the city of Hangzhou, China, to examine BPS and five kinds of Clx-BPSs. The goals of the current study were to assess the presence of Clx-BPSs in collected indoor dust samples and their relationships in concentration levels with BPS and estimate the amount of human intake of BPS and Clx-BPSs through indoor dust inhalation. This study is the first to demonstrate the existence of Clx-BPSs in indoor dust, which is vital for assessing potential risks associated with human exposure to Clx-BPSs.

## 2. Materials and Methods

### 2.1. Standard Chemicals and Reagents

Certified standard chemicals of BPS (purity 98%) and ^13^C_12_-BPS (99%) were purchased from Wellington Laboratories (Guelph, Canada). Certified standards of Cl_1_-BPS, Cl_2_-BPS-1, 2-chloro-4-(3-chloro-4-hydroxyphenyl)sulfonylphenol (Cl_2_-BPS-2), 2,6-dichloro-4-(3-chloro-4-hydroxyphenyl)sulfonylphenol (Cl_3_-BPS), and 2,6-dichloro-4-(3,5-dichloro-4-hydroxyphenyl)sulfonylphenol (Cl_4_-BPS) were obtained from Toronto Research Chemicals (North York, NY, USA). Full names, abbreviations, and CAS numbers of BPS and Clx-BPSs are delineated in the Supplementary Materials.

HPLC-grade solvents (including pure acetonitrile, methanol, and pure water) were obtained from Merck KGaA (Darmstadt, Germany). Aqueous ammonia (28–30% NH_3_ basis), formic acid, anhydrous sodium sulfate, and ammonium acetate were from Sigma Co. (Shanghai, China). 

### 2.2. Indoor Dust Collection

During June–August 2022, we conducted a comprehensive sampling campaign to collect indoor dust samples from diverse residential apartments (comprising 178 buildings) in Hangzhou City, China. These sampling sites were distributed across both suburban (Fuyang district and Lin’an district) and urban (Xihu district and Gongshu district) regions of Hangzhou City, as illustrated in the Supplementary Materials, Figure S1. The weather during the sampling period was clear and sunny, with the air temperature and humidity being 25–30 °C and 40–60%RH, respectively. For indoor dust sampling, disposable bristle brushes were employed to sweep the bedroom, kitchen, and living room floors, following previous studies [35,36,37]. The floors of these rooms were covered in ceramic tile or wood. In each residential apartment, 8–15 g of indoor dust sample was obtained. In order to obtain a representative indoor dust sample, each sample comprised a composite mixture of 2–4 subsamples collected from the same residential apartment within seven days. In total, 178 indoor dust samples were taken from the Fuyang (*n* = 43), Lin’an (*n* = 47), Xihu (*n* = 40), and Gongshu (*n* = 48) districts. These indoor dust specimens were individually wrapped using clean aluminum foil and then stored in the freezer at −60 °C. Additionally, as a quality control measure, field blank samples, each consisting of 5 g anhydrous Na_2_SO_4_, were transported alongside the real indoor dust samples.

### 2.3. Indoor Dust Sample Extraction

Each indoor dust sample was individually dried under vacuum, ground, and passed through an 80-mesh sieve. After that, these samples were treated, according to previous studies [38,39,40,41]. In brief, the extraction procedure began by transferring dried indoor dust samples (2.0 g) into 15 mL glass tubes and adding mass-labeled internal standards (2.0 ng each). Next, 6 mL of 90%/10% methanol/water was transferred into the tubes, which were then vortexed for 1 min. Following this, these mixtures underwent agitation at 200 rpm for 30 min, followed by sonication at 435 MHz for 40 min, and centrifugation at 5000× *g* for 8 min. The resulting supernatant solutions were then separated. The remaining sample residue underwent a secondary extraction with 6 mL of the 90%/10% methanol/water solution. The two obtained supernatants were combined and passed through a CNWBOND carbon-GCB SPE cartridge (6 mL, 500 mg; ANPEL; Shanghai, China), which had been preconditioned with 10 mL of methanol. Subsequently, the purified extracts were dried to remove the solvent residue using high-purity N_2_ gas. Finally, the residual material was reconstituted with a 50:50 (*v*/*v*) methanol/water solution (50 μL).

### 2.4. Instrumental Analysis

Chromatographic separation of BPS and individual Clx-BPSs was performed using a high-performance liquid chromatography (UltiMate™ 3000; Thermo Co., New York, NY, USA), with a C_18_ chromatographic column (Hypersil GOLD™; 100 mm × 2.1 mm, 3 μm particle size; Thermos-Fisher, Shanghai, China). The mobile phase used for gradient elution was composed of 0.1% (*v*/*v*) aqueous ammonia in pure water (solvent A) and pure methanol (solvent B). The gradient conditions of the mobile phase were as follows: 0.0–0.5 min, 10% B; 0.5–1.5 min, 10–40% B; 1.5–10 min, 90% B; 10–12 min, 90% B, followed by a return to 10% in 0.1 min. The post-delay time of 4 min was implemented to recondition the C_18_ column with 10% B. The tandem mass spectrometer (Q Exactive; Thermo-Fisher, New York, NY, USA) was carried out in the multiple reaction monitoring (MRM) mode. The electrospray ionization was carried out in the negative ion mode. The mass spectrometric parameters were individually optimized for each compound. MRM transition information of target analytes is described in the Supplementary Materials (Table S2). 

### 2.5. QA/QC

Organic solvents utilized for sample extraction underwent scrutiny to detect any traces of BPS and Clx-BPS contamination prior to their application. Following the analysis of every batch of 10 samples, a procedural blank was examined. In order to monitor the potential carry-over and instrumental background pollution during instrumental analysis, a pure solvent (methanol, 10 μL) was measured after every 10 samples. Glassware was employed throughout the sample collection and extraction procedures to prevent any contamination of BPS.

The quantification of analyte concentrations present in the sample extracts was performed using the internal standard method. Calibration curves spanning six concentration levels were constructed for individual target analytes, exhibiting linearity with correlation coefficients (R-squared) exceeding 0.995. BPS and Clx-BPSs were not detected in any procedural blank samples. So, the limits of detection (LODs) for BPS and Clx-BPSs were determined by calculating the analyte levels that yielded a signal-to-noise ratio of three. In this study, the calculated LODs of BPS and Clx-BPSs ranged from 0.012 (Cl_2_-BPS-1) to 0.039 (BPS) μg/g in indoor dust. We evaluated extraction recoveries of BPS and Clx-BPSs via analyzing indoor dust samples fortified with target analytes (0.050, 0.20, or 5.0 μg/g; *n* = 5), with the subtraction of background concentrations of the analytes. Extraction of BPS and Clx-BPSs in the indoor dust matrix displayed a recovery of 80–107% (Supplementary Materials, Table S3). The precision of the employed analytical approach was assessed by determining the relative standard deviation (RSD) of results obtained at three distinct concentration levels. The intra-day RSD (*n* = 5) of the quantified concentrations in indoor dust ranged from 4.7 to 13%. Additionally, the inter-day RSD (*n* = 5) of the calculated BPS and Clx-BPS concentrations in indoor dust samples, evaluated over a one-week period, did not exceed 18%. 

### 2.6. Estimation of Daily Intake

To estimate the amount of human ingestion of BPS and Clx-BPSs through the indoor dust inhalation pathway, the daily intake (DI; ng/kg bw/day) was calculated according to the following formula [42]:

$$DI=\frac{{1000\times C}_{dust}\times IR}{BW}$$

where DI means the general population’s daily intake of BPS and Clx-BPSs through indoor dust inhalation. *C*_dust_ represents measured levels of BPS and Clx-BPSs in indoor dust, expressed in μg/g. *IR* denotes the indoor dust ingestion rate, which is assigned values of 0.02 g/day for infants, 0.05 g/day for children, and 0.08 g/day for adults [39,40,43]. *BW* signifies the human body weight, with assumed values of 5.0 kg for infants, 29 kg for children, and 63 kg for adults [44,45].

### 2.7. Statistical Analysis

The statistical analysis was performed based on the SPSS^®^ Statistics software version 29 (IBM, Charleston, SC, USA). BPS and Clx-BPSs exhibiting detection frequencies below 50% in indoor dust samples were excluded from statistical analysis. For analytes with detection frequencies surpassing 50%, concentrations falling below the LODs were imputed as LODs/√2. Spearman’s rank correlation coefficient (*r*_s_) was calculated to explore associations among concentrations of BPS and various Clx-BPSs in indoor dust samples. Mann–Whitney *U* test was employed to evaluate the distinction in levels of BPS and Clx-BPSs in indoor dust samples between urban and suburban areas. Differences in the concentrations of BPS and Clx-BPSs detected in indoor dust from urban or suburban areas were compared on the basis of the Mann–Whitney *U* test. 

## 3. Results and Discussion

### 3.1. Occurrence of BPS in Indoor Dust

BPS was detectable in 94% of collected indoor dust (*n* = 178) samples, with an average concentration of 0.63 μg/g (median 0.52 μg/g, <LD–2.4 μg/g) (Table 1). Previous studies had consistently reported the high detection frequency (>75%) of BPS in other Chinese indoor dust [46,47]. Comparatively, the average BPS level reported here is greater, relative to that reported in indoor dust from the dormitory of Chinese students (0.13 μg/g, <LD–0.81 μg/g) [46] and Korean homes (0.20 μg/g, 0.037–0.51 μg/g) [48], but lower than that from Japan (1.7 μg/g) and the United States (1.5 μg/g) [43]. 

In addition, the indoor dust samples from the Xihu (average 0.75 μg/g) and Gongshu districts (0.71 μg/g; urban regions) contained higher concentrations of BPS than those from the Fuyang (0.42 μg/g) and Lin’an districts (0.61 μg/g; suburban regions) (Table S4). This is possibly related to the higher consumption rates of plastic containers, food packaging, and thermal paper in urban residential apartments. The increased utilization of electrical and electronic devices, as well as plastic furniture, may also lead to higher levels of BPS in indoor dust samples collected from urban residential houses [18,25,49]. Alternatively, the air pollution in Hangzhou’s urban areas is more severe than that in the urban areas, with possibly higher levels of BPS in atmospheric particulate matter. Consequently, a greater amount of BPS may settle indoors via atmospheric particulate matter deposition, resulting in higher levels of BPS in urban indoor dust.

### 3.2. Chlorinated Derivatives of BPS in Indoor Dust

**Figure 1:**
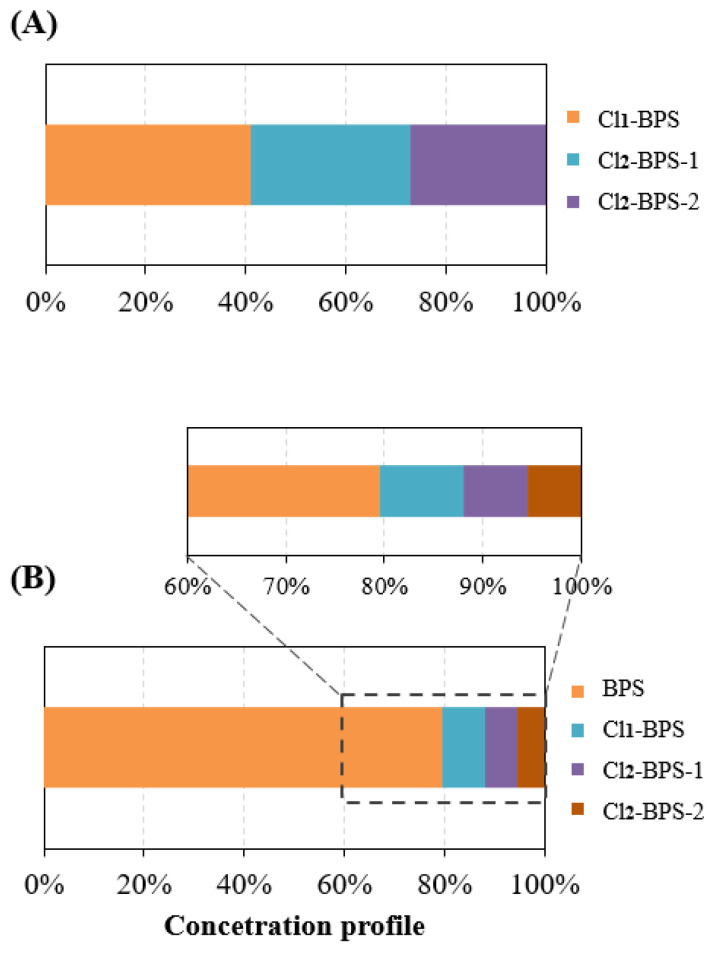
(**A**) Concentration profiles of Clx-BPSs in indoor dust samples from Hangzhou City, China. (**B**) Concentration profiles of BPS and Clx-BPSs in indoor dust samples from Hangzhou City, China.

Monitoring results exhibited that all five target Clx-BPSs compounds were detected in Hangzhou indoor dust samples (*n* = 178; Table 1). Only 12 indoor dust samples did not contain any detectable Clx-BPSs. The concentration of total detected Clx-BPSs (∑Clx-BPSs) was in the range of <LD—2.4 μg/g (average 0.72 μg/g, median 0.66 μg/g). Among the detected Clx-BPSs, Cl_1_-BPS was the most frequently found homologue (detection frequency 70%), followed by Cl_2_-BPS-2 (65%) and Cl_2_-BPS-1 (61%). Cl_3_-BPS and Cl_4_-BPS were much less frequently detected, with the detection frequency of 31% and 18%, respectively. Consistently, Cl_1_-BPS was the most abundant analyte, displaying an average level of 0.048 μg/g (<LD—0.24 μg/g), followed by Cl_2_-BPS-1 (0.035 μg/g, <LD—0.14 μg/g) and Cl_2_-BPS-2 (0.031 μg/g, <LD—0.13 μg/g). The concentrations of Cl_1_-BPS accounted for an average of 41% of ∑Clx-BPSs in indoor dust (Figure 1). Spatially, urban indoor dust from Hangzhou City contained relatively higher concentrations of Cl_1_-BPS, Cl_2_-BPS-1, and Cl_2_-BPS-2 compared with that from the suburban districts. For instance, the mean concentrations of Cl_1_-BPS, Cl_2_-BPS-1, and Cl_2_-BPS-2 in indoor dust from the Xihu district were 0.066 μg/g, 0.046 μg/g, and 0.033 μg/g, respectively, compared with 0.020 μg/g, 0.016 μg/g, and 0.020 μg/g, respectively, in indoor dust from the Fuyang district. This concentration trend is generally consistent with that observed for BPS. The potential toxic effects of exposure to these detected Clx-BPSs through the inhalation of indoor dust on human health may include oxidative stress in the lungs, systemic inflammation, and allergic respiratory diseases [50]. These potential health risks are concerning, especially for children and infants, who are more susceptible to indoor dust pollutants because of higher ingestion rates and sensitivity. These detected Clx-BPSs may disrupt endocrine functions and are linked to health issues such as obesity and developmental defects [51]. Economically, cities may face increased healthcare costs and productivity losses because of the health impacts on the population. Additionally, addressing indoor dust pollution requires substantial public health interventions and regulatory measures, which could strain municipal resources. This study underscores the need for comprehensive strategies to mitigate indoor dust pollution of these detected Clx-BPSs and protect public health.

**Figure 2:**
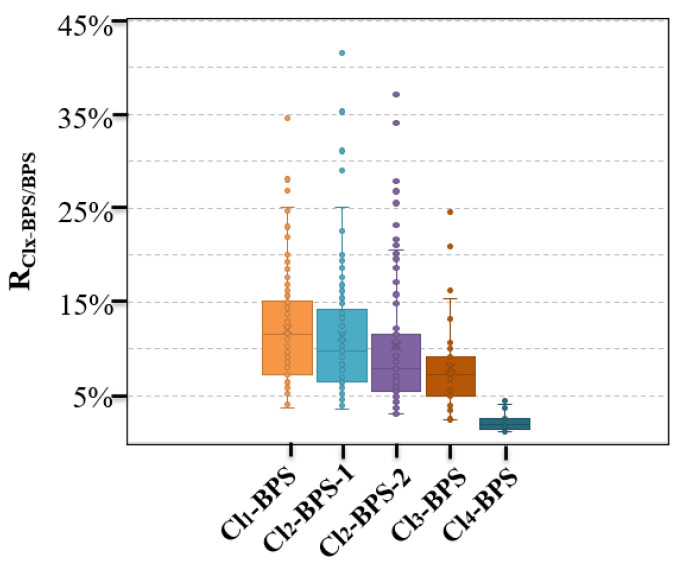
Concentration ratios (R_Clx-BPS/BPS_) of individual Clx-BPSs to BPS in indoor dust from Hangzhou, China.

This is the first study reporting the existence and concentration levels of Clx-BPSs in indoor dust samples. A previous study already analyzed many paper products from China [18] and reported that Clx-BPSs were primarily detectable in thermal paper, corrugated boxes, mailing envelopes, and newspaper samples, with concentrations of <LD—3.76 μg/g, <LD—30.2 ng/g, <LD—31.0 ng/g, and<LD—98.5 ng/g, respectively. Yang et al. merely determined the existence and levels of Cl_1_-BPS in thermal paper (detection frequency 5.9%, 0.27–2.5 μg/g) and non-thermal paper (42%, 0.10–10 ng/g) samples [19]. A limited number of studies have been conducted to monitor the presence of Clx-BPSs in other environmental matrices apart from these two studies. Notably, this study focused exclusively on investigating indoor dust specimens collected from China. As the existence of Clx-BPSs was confirmed in a majority of the collected indoor dust samples, the potential for global pollution from these compounds should be a matter of significant concern. In addition, we calculated and reported the ratios of indoor dust concentrations of individual Clx-BPSs to BPS (R_Clx-BPS/BPS_) (Figure 2). The average R_Clx-BPS/BPS_ ranged from 2.2 (Cl_4_-BPS) to 12% (Cl_1_-BPS) in indoor dust, which is comparatively lower than that reported in various paper products (0.057–6.1%), such as food contact paper and thermal paper products [18]. This may suggest that the degree of chlorination of BPS was higher in indoor dust than in reported paper products.

**Figure 3:**
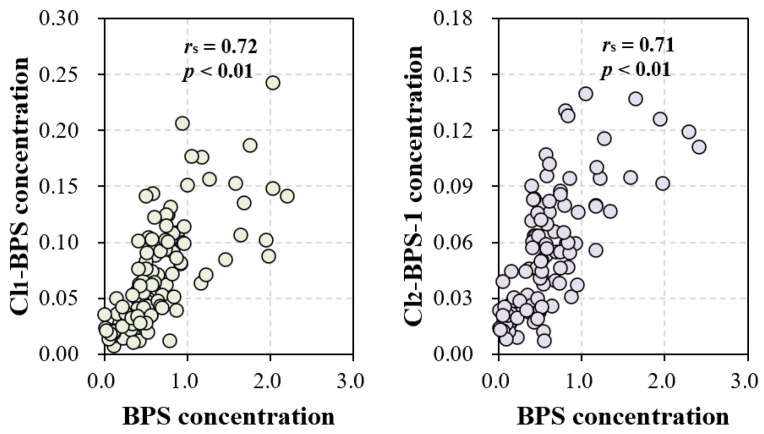
Correlations among concentrations of BPS, Cl_1_-BPS, and Cl_2_-BPS-1 in all of the indoor dust samples.

The exact sources of Clx-BPSs occurring in the indoor dust are currently unknown. In this study, we observed significant correlations in indoor dust concentrations between BPS and Cl_1_-BPS (*r*_s_ = 0.72, *p* < 0.01) and also between BPS and Cl_2_-BPS-1 (*r*_s_ = 0.71, *p* < 0.01; Figure 3 and Table S5). Significant correlations were barely observed among various Clx-BPSs, such as between monochloro-BPS and dichloro-BPS. Moreover, concentration levels of BPS quantified in indoor dust are much higher than that of individual Clx-BPSs (Figure 1). For instance, the average indoor dust concentration of BPS is around 13 times greater relative to that of Cl_1_-BPS. These data suggest that the elevated Cl_2_-BPS-1 and Cl_1_-BPS levels may result from the chlorination of BPS or that BPS and Cl_2_-BPS-1 (or Cl_1_-BPS) have similar sources in indoor dust. Previous studies have demonstrated the generation of Clx-BPSs during the chlorine bleaching of base paper that contained BPS [18,52]. We speculate that the BPS residue in indoor dust may serve as a precursor for the generation of Clx-BPSs. For example, BPS occurring in indoor dust may react with chlorine-containing household cleaning products to form Clx-BPSs, similar to findings reported for BPA [53]. Alternatively, Clx-BPSs may be applied as industrial additives or intermediates to produce consumer products. In addition, the type of indoor environment may significantly influence the occurrence of BPS and Clx-BPSs in indoor dust, thereby affecting their human exposure levels. Various factors, including the types of materials and products used within indoor spaces, the presence of electronic devices, and the level of urbanization, play crucial roles in determining the indoor dust concentrations of BPS and Clx-BPSs. 

### 3.3. Estimated Intake of BPS and Clx-BPSs

The average levels of DIs for BPS among infants, children, and adults living in the urban area were 3.0, 1.3, and 0.94 ng/kg bw/day, respectively, which are higher than that in the suburban area (2.1, 0.90, and 0.66 ng/kg bw/day, respectively) (Table 2). The DI of individual Clx-BPSs was much lower than that of BPS. Among the Clx-BPSs homologues, Cl_1_-BPS had the highest average DI (0.12–0.38 ng/kg bw/day), followed by Cl_2_-BPS-1 (0.085–0.27 ng/kg bw/day) and Cl_2_-BPS-2 (0.066–0.21 ng/kg bw/day), for target people living in the urban area. While Cl_2_-BPS-2 (0.048–0.15 ng/kg bw/day) had slightly elevated average DIs compared with Cl_2_-BPS-1 (0.045–0.14 ng/kg bw/day) for target people living in the urban area. Moreover, infants exhibited significantly elevated (*p* < 0.05) DIs of BPS and Clx-BPSs compared with children and adults, which is possibly attributable to their higher rates of indoor dust ingestion and lower body weights and also to the different indoor environments. For instance, BPS and Clx-BPSs for infants residing in the urban area had average DI values of 0.21–3.0 ng/kg bw/day, while those for children and adults were markedly lower at 0.089–1.3 and 0.066–0.94 ng/kg/day, respectively. 

**Figure 4:**
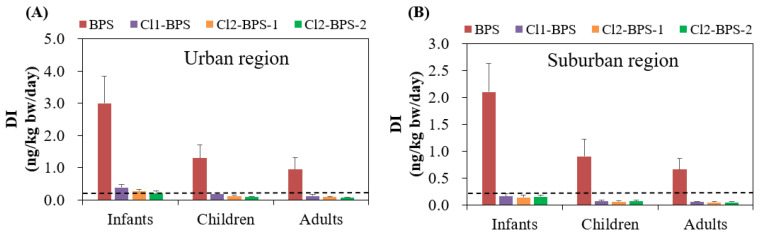
Estimated daily intake (ng/kg bw/day; mean ± SD) of BPS and Clx-BPSs through indoor dust ingestion for the general population in the (**A**) urban and (**B**) suburban regions. The dashed line means the TDI value of BPA set by the European Food Safety Authority.

Since no studies have reported tolerable daily intakes (TDIs) of various Clx-BPSs for the general population, the TDI established for the structurally similar BPA was utilized as an alternative to assess the health risk associated with human Clx-BPSs and BPS exposure. Considering that the TDI value of BPA set by the European Food Safety Authority was 0.2 ng/kg bw/day [54], the average DIs of Clx-BPSs proposed for infants living in the urban area (0.21–0.38 ng/kg/day) were greater than this threshold (Figure 4). This result suggests that human Clx-BPS exposure through indoor dust intake could possibly result in toxicity concerns. However, this speculation should be interpreted with caution since TDI of BPA was used as the threshold. Han et al. reported that individuals exposed to Clx-BPSs through handling the thermal paper also surpassed the TDI of BPA [18], which may potentially pose significant adverse impacts on human health.

## 4. Conclusions

The residue of BPS in environmental matrices has been extensively studied, but there is limited knowledge regarding Clx-BPSs. This study first reveals the presence of various Clx-BPSs in Chinese indoor dust samples. Five target Clx-BPSs were identified in collected Chinese indoor dust specimens despite their varying detection frequencies. Significant correlations in concentrations were observed between BPS and Cl_1_-BPS, as well as between BPS and Cl_2_-BPS-1, suggesting that chlorination of BPS may represent a source of certain indoor dust Clx-BPSs. The origins of Clx-BPSs in indoor dust should be elucidated in future studies. Urban indoor dust contained relatively higher concentrations of BPS and Clx-BPSs compared with that from the suburban districts. Utilizing the quantified levels of indoor dust Clx-BPSs in the current study, the daily consumption of Clx-BPSs through the ingestion of indoor dust was assessed for infants, children, and adults. Furthermore, other sources of human exposure to Clx-BPSs require more investigation. In addition, further work is warranted in order to assess the impact of simultaneous exposure to BPS and Clx-BPSs on humans.

## Figures and Tables

**Table 1:** Concentrations (μg/g) and detection frequencies (DF; %) of BPS and chlorinated derivatives of BPS in indoor dust samples (*n* = 178).

	Detection Frequency	Average	Percentile				
			Minimum	25th	Median	75th	Maximum
BPS	94%	0.63	<LD	0.17	0.52	0.80	2.4
Cl_1_-BPS	70%	0.048	<LD	<LD	0.055	0.099	0.24
Cl_2_-BPS-1	61%	0.035	<LD	<LD	0.046	0.076	0.14
Cl_2_-BPS-2	65%	0.031	<LD	<LD	0.037	0.058	0.13
Cl_3_-BPS	31%	NC	<LD	<LD	<LD	0.055	0.090
Cl_4_-BPS	18%	NC	<LD	<LD	<LD	<LD	0.057

Note that NC means not calculated.

**Table 2:** Estimated daily intake (ng/kg bw/day) of BPS and Clx-BPSs through indoor dust ingestion for the general population.

	Infants				Children				Adults			
	Average	Median	Min	Max	Average	Median	Min	Max	Average	Median	Min	Max
Urban region												
BPS	3.0	2.7	<0.021	9.6	1.3	1.2	<0.0086	4.2	0.94	0.87	<0.0063	3.1
Cl_1_-BPS	0.38	0.37	<0.031	0.97	0.17	0.16	<0.0095	0.42	0.12	0.12	<0.0073	0.31
Cl_2_-BPS-1	0.27	0.25	<0.028	0.56	0.12	0.11	<0.012	0.24	0.085	0.080	<0.0090	0.18
Cl_2_-BPS-2	0.21	0.20	<0.034	0.38	0.089	0.085	<0.015	0.16	0.066	0.062	<0.011	0.12
Cl_3_-BPS	-	-	<0.051	0.36	-	-	<0.011	0.15	-	-	<0.0082	0.11
Cl_4_-BPS	-	-	<0.027	0.14	-	-	<0.0094	0.060	-	-	<0.0069	0.044
Suburban region												
BPS	2.1	1.6	<0.021	9.2	0.90	0.70	<0.0086	3.9	0.66	0.52	<0.0063	2.9
Cl_1_-BPS	0.16	0.13	<0.031	0.43	0.068	0.057	<0.0095	0.18	0.050	0.042	<0.0073	0.14
Cl_2_-BPS-1	0.14	0.095	<0.028	0.55	0.062	0.041	<0.012	0.24	0.045	0.030	<0.0090	0.17
Cl_2_-BPS-2	0.15	0.11	<0.034	0.55	0.066	0.047	<0.015	0.24	0.048	0.035	<0.011	0.18
Cl_3_-BPS	-	-	<0.051	0.23	-	-	<0.011	0.10	-	-	<0.0082	0.072
Cl_4_-BPS	-	-	<0.027	0.23	-	-	<0.0094	0.10	-	-	<0.0069	0.073

Note that—means not calculated.

## Data Availability

Data will be available on request.

## Supplementary Materials

The following supporting information can be downloaded at: https://www.mdpi.com/article/10.3390/toxics12070448/s1. Figure S1: Map of the suburban (Fuyang district and Lin’an district) and urban (Xihu district and Gongshu district) areas in Hangzhou, China. Table S1: Abbreviations, Full Names, and CAS Numbers of the Target Analytes; Table S2: MRM Transitions and Collision Energy of the Target Analytes; Table S3: Limits of Detection (LODs) and Extraction Recoveries of the Target Analytes in Indoor Dust; Table S4: Concentrations (μg/g) of BPS and Clx-BPSs in Indoor Dust from Different Areas of the Hangzhou City. NC Means Not Calculated; Table S5: Correlations Among Concentrations of BPS and Clx-BPSs in Indoor Dust from Hangzhou, China.
