# Supplementary material for: Bisphenol S and Its Chlorinated Derivatives in Indoor Dust and Human Exposure
Source: Toxics. 2024 Jun 21;12(7):448. doi: 10.3390/toxics12070448 (PMC11280507; doi:10.3390/toxics12070448)
Supplement: Supplementary file 1 [file toxics-12-00448-s001.zip › toxics-3040694-supplementary.pdf]

SUPPORTING INFORMATION

Bisphenol S and Its Chlorinated Derivatives in Indoor Dust and Human Exposure

Yi Qian[1]; Jianqiang Zhu[1]; Ruyue Guo[2]; Hangbiao Jin[2,*]

[1] Department of Environmental Engineering, Taizhou University, Taizhou, Zhejiang 318000, P. R. China.

[2] Key Laboratory of Microbial Technology for Industrial Pollution Control of Zhejiang Province, College of Environment, Zhejiang University of Technology, Hangzhou, Zhejiang 310032, P. R. China.

Table S1. Abbreviations, Full Names, and CAS Numbers of the Target Analytes.

Full name	Abbreviation	CAS number
**2-Chloro-4-((4-hydroxyphenyl)sulfonyl)phenol**	Cl_1-BPS	80396-77-6
**2,6-Dichloro-4-(4-hydroxyphenyl)sulfonylphenol**	Cl_2-BPS-1	61779-46-4
**2-Chloro-4-(3-chloro-4-hydroxyphenyl)sulfonylphenol**	Cl_2-BPS-2	80-07-9
**2,6-Dichloro-4-(3-chloro-4-hydroxyphenyl)sulfonylphenol**	Cl_3-BPS	64485-93-4
**2,6-Dichloro-4-(3,5-dichloro-4-hydroxyphenyl)sulfonylphenol**	Cl_4-BPS	77398-70-4
**Bis(4-hydroxyphenyl) sulfone**	BPS	80-09-1
**$^{13}C_{12}$-Bisphenol S**	$^{13}C_{12}$-BPS	

Table S2. MRM Transitions and Collision Energy of the Target Analytes.

	MRM transition			
**Abbreviation**	**Quantification (*m/z*)**	**Collision energy (eV)**	**Qualification (*m/z*)**	**Collision energy (eV)**
**Cl_1-BPS**	283/126	22	283/142	28
**Cl_2-BPS-1**	317/160	26	317/176	24
**Cl_2-BPS-2**	317/126	25	317/142	20
**Cl_3-BPS**	351/176	25	351/160	25
**Cl_4-BPS**	385/176	21	385/160	20
**BPS**	249/108	30	249/156	25
**$^{13}C_{12}$-BPS**	261/114	25		

Table S3. Limits of Detection (LDs) and Extraction Recoveries of the Target Analytes in Indoor Dust.

		Extraction recovery (%)					
		Spiked at 0.050 μg/g		Spiked at 0.20 μg/g		Spiked at 5.0 μg/g	
	LODs (ng/g)	Mean	SD	Mean	SD	Mean	SD
**Cl_1-BPS**	2.0	91	7	94	6	104	11
**Cl_2-BPS-1**	1.2	89	9	88	4	101	7
**Cl_2-BPS-2**	1.4	96	10	96	10	107	4
**Cl_3-BPS**	2.7	88	9	99	6	83	9
**Cl_4-BPS**	1.9	100	7	80	9	86	4
**BPS**	3.9	82	10	100	4	90	5

Table S4. Concentrations (μg/g) of BPS and Clx-BPSs in Indoor Dust from Different Areas of the Hangzhou City. NC Means Not Calculated.

	Detection frequency	Mean	Median	Range
**Xihu district**				
**BPS**	100%	0.75	0.71	0.36–1.8
**Cl_1-BPS**	70%	0.066	0.063	< LOD–0.19
**Cl_2-BPS-1**	65%	0.046	0.043	< LOD–0.13
**Cl_2-BPS-2**	60%	0.033	0.031	< LOD–0.091
**Cl_3-BPS**	50%	0.024	0.028	< LOD–0.073
**Cl_4-BPS**	38%	NC	< LOD	< LOD–0.027
**Gongshu district**				
**BPS**	98%	0.71	0.67	< LOD–2.4
**Cl_1-BPS**	69%	0.068	0.062	< LOD–0.24
**Cl_2-BPS-1**	65%	0.046	0.050	< LOD–0.14
**Cl_2-BPS-2**	58%	0.035	0.020	< LOD–0.095
**Cl_3-BPS**	29%	NC	< LOD	< LOD–0.090
**Cl_4-BPS**	38%	NC	< LOD	< LOD–0.035
**Fuyang district**				
**BPS**	86%	0.42	0.224	< LOD–2.2
**Cl_1-BPS**	65%	0.020	0.016	< LOD–0.099
**Cl_2-BPS-1**	63%	0.016	0.012	< LOD–0.056
**Cl_2-BPS-2**	67%	0.020	0.013	< LOD–0.11
**Cl_3-BPS**	14%	NC	< LOD	< LOD–0.041
**Cl_4-BPS**	4.7%	NC	< LOD	< LOD–0.025
**Lin'an district**				
**BPS**	94%	0.61	0.461	< LOD–2.3
**Cl_1-BPS**	77%	0.036	0.033	< LOD–0.107
**Cl_2-BPS-1**	51%	0.030	0.013	< LOD–0.14
**Cl_2-BPS-2**	72%	0.037	0.027	< LOD–0.13
**Cl_3-BPS**	21%	NC	< LOD	< LOD–0.057
**Cl_4-BPS**	23%	NC	< LOD	< LOD–0.057

Table S5. Correlations Among Concentrations of BPS and Clx-BPSs in Indoor Dust from Hangzhou, China.

		Cl_1-BPS	Cl_2-BPS-1	Cl_2-BPS-2	Cl_3-BPS	Cl_4-BPS
**BPS**	**Spearman Correlation**	.722**	.714**	.244	.217	.154
	**Sig. (2-tailed)**	.000	.000	.109	.112	.400
	n	121	102	107	55	49
**Cl_1-BPS**	**Spearman Correlation**	1	.169	.173	.201	.317
	**Sig. (2-tailed)**		.0190	.320	.073	.073
	n		75	88	39	41
**Cl_2-BPS-1**	**Spearman Correlation**		1	.265	.240	.262
	**Sig. (2-tailed)**			.049	.030	.127
	n			73	28	33
**Cl_2-BPS-2**	**Spearman Correlation**			1	.149	.165
	**Sig. (2-tailed)**				.447	.402
	n				32	28
**Cl_3-BPS**	**Spearman Correlation**				1	.208
	**Sig. (2-tailed)**					.352
	n					22

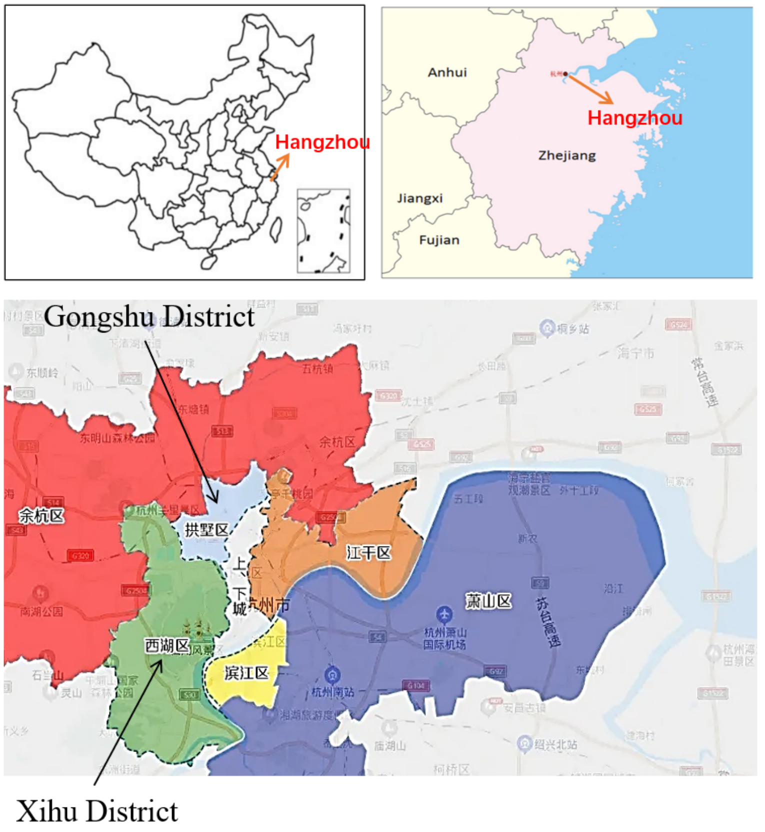

Figure S1. Map of the suburban (Fuyang district and Lin'an district) and urban (Xihu district and Gongshu district) areas in Hangzhou, China.
